# Feeding ecology and diet of the southern geladas (*Theropithecus gelada obscurus*) in human‐modified landscape, Wollo, Ethiopia

**DOI:** 10.1002/ece3.7927

**Published:** 2021-07-22

**Authors:** Zewdu Kifle, Afework Bekele

**Affiliations:** ^1^ Department of Biology Bahir Dar University Bahir Dar Ethiopia; ^2^ Department of Zoological Sciences Addis Ababa University Addis Ababa Ethiopia

**Keywords:** conservation, dietary flexibility, Ethiopia, fruits, human‐modified landscape, southern gelada

## Abstract

Studying the dietary flexibility of primates that live in human‐modified environments is crucial for understanding their ecological adaptations as well as developing management and conservation plans. Southern gelada (*Theropithecus gelada obscurus*) is an endemic little‐known subspecies of gelada that inhabits human‐modified landscapes in the northern central highlands of Ethiopia. During an 18‐month period, we conducted this intensive study in an unprotected area of a human‐modified landscape at Kosheme in Wollo to investigate the feeding ecology of southern geladas and their dietary responses to seasonal variations. We quantified the monthly and seasonal diet data from a band of southern geladas using instantaneous scan sampling method at 15‐min intervals, and green grass phenology and availability using visual inspection from the randomly selected permanent plots. The overall average diet of southern geladas at Kosheme constituted grass blades 55.4%, grass undergrounds 13.2%, grass bulbs 5.6%, grass seeds 5.4%, herb leaves 4.0, fruits 7.3%, and cereal crops 5.6%. Grass blade consumption increased with increasing green grass availability, while underground food consumption increased with decreasing green grass availability, and vice versa. Southern geladas spent significantly more time feeding on the grass blades and herb leaves and significantly less time on bulbs during the wet season than the dry season. Underground grass items (rhizomes and corms) were not consumed during the wet season, but made up 22.3% of the dry season diet. Thus, although grass blades are staple diet items for geladas, underground diet items are important “fallback foods” at Kosheme. Our result shows insights into the dietary flexibility southern geladas adopt to cope with human‐modified landscapes of the north‐central Ethiopian Highlands. Thus, the study contributes to a better understanding of how changing environments shape primate ecology and evolution.

## Graphical abstract

Studying the dietary flexibility of primates that live in human‐modified environment is crucial for understanding ecological adaptations. We conducted this intensive study in an unprotected area of human‐modified landscape to investigate the dietary responses of southern geladas to human disturbances and seasonal variations. The study contributes to a better understanding of how changing environments shape primate ecology and evolution.

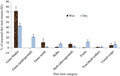

## 1 INTRODUCTION

Habitat loss due to deforestation and land conversion are major causes of the decline of many primate species (Chapman & Peres, 2001; Estrada, 2013; Irwin, 2016). Although the number of protected areas in many countries has increased over the recent decades, reserves are insufficient to harbor all primate populations (Estrada et al., 2012). Thus, many primate species live in human‐dominated landscapes outside protected areas by sharing resources with the local people (Bryson‐Morrison et al., 2017; Hockings & McLennan, 2012; Isabirye‐Basuta & Lwanga, 2008; Lee, 2010; Mekonnen et al., 2020; Strum, 2010). Agricultural expansion and land degradation are the main threats of many primate species inhabiting unprotected landscapes across East Africa (Chapman & Peres, 2001; Cowlishaw & Dunbar, 2000; Fedigan & Jack, 2001; Kifle & Bekele, 2020a, 2020b). Primates that inhabit such human‐modified areas face numerous socio‐ecological as well as demographic constraints (Fuentes & Hockings, 2010; Hockings et al., 2012). Human‐modified landscapes reduce the habitat quality that can cause changes in primate feeding behavior and dietary diversity (Campbell‐Smith et al., 2011; Guzmán et al., 2016; Ménard et al., 2014; Pozo‐Montuy et al., 2013; Riley, 2007; Singh et al., 2001; Wong et al., 2006). As preferred foods are commonly lacked in the human‐modified landscapes, primates fed on less preferred and diversified diet items (Chaves et al., 2012; Dela, 2007; Dunn et al., 2012).

In response to habitat changes, primates can develop ecological and behavioral flexibility (Arroyo‐Rodríguez & Fahrig, 2014; Mekonnen et al., 2018; Melzer et al., 2014; Onderdonk & Chapman, 2000). They can become flexible in their feeding ecology and other socio‐ecological behaviors to suit themselves in the human‐altered landscapes (Arroyo‐Rodríguez & Fahrig, 2014; Campbell‐Smith et al., 2011; Chaves et al., 2012; Guzmán et al., 2016; Mekonnen et al., 2018; Ménard et al., 2014; Pozo‐Montuy et al., 2013). In addition, habitat degradation decreases food availability for primate species inhabiting those areas (Arroyo‐Rodríguez & Mandujano, 2006). Decrease in food availability and habitat size may lower the carrying capacity of the environment, which in turn can result in primate population declines or local extirpation (Chapman et al., 2010; Chapman et al., 2016).

Different primate species show variable responses to human disturbance environments (Bryson‐Morrison et al., 2017). Understanding the feeding response of primates to landscape changes has received attention by primatologists only in the last two decades (Arroyo‐Rodríguez & Fahrig, 2014). Long‐term ecological studies on primate populations that live in human‐modified landscapes are crucial to improve our knowledge on the capacity of primates to adapt to habitat disturbances (Chapman & Peres, 2001; Corlett, 2011; Hill, 2017; Mekonnen et al., 2017; Struhsaker, 2008). It is also a necessary precursor to primate conservation programs (Marsh, 2003; Mekonnen et al., 2017).

In addition, to understand the dietary adaptability of primates to human disturbances, not only the contents of the species' diet, but also the seasonal variation in the diet should be examined. Primates may consume low‐quality “fallback foods” to deal with temporal variation in food availability by using different foraging strategies (Jarvey et al., 2018; Marshall et al., 2009; Marshall & Wrangham, 2007). Thus, as a result of seasonal variation in food availability, primates may switch consumptions from one diet item to another to optimize nutrient intake (Jarvey et al., 2018).

Seasonal fluctuations in environmental variables influence food availability of animal's diet (Chouteau, 2006). This in turn influences animal's diet choices. Dietary shifts typically correspond with seasonal resource scarcity (Hanya, 2004; Yiming, 2006). Rainfall is a major determinant of plant productivity, where seasonal patterns correspond with resource availability. Variation in food availability is one of the main factors determining seasonal variation in the diet of primates (Fashing et al., 2014; Hanya, 2004; Jarvey et al., 2018; McConkey et al., 2003). For example, primates inhabiting tropical forests often ingest mature leaves and unripe fruits during lean‐seasons when preferred foods are scarce (Chapman & Rothman, 2009; Marshall et al., 2009). During the rainy season, when ripe fruit was scarce, chimpanzees relied heavily on piths and leaves (Basabose, 2002). Colobine species such as Francois' langurs (*Trachypithecus francoisi*) feed on more low‐quality, subsistence foods, such as petioles and stems, when high‐quality foods, such as young leaves, were scarce (Zhou et al., 2006). Geladas (*Theropithecus gelada*) in the Simien Mountains National Park spent considerable time consuming underground food items in the dry season (Hunter, 2001; Jarvey et al., 2018).

Southern geladas (*Theropithecus gelada obscurus*) are an endemic subspecies of gelada that live in human‐modified landscape across the northern central highlands of Ethiopia. Geladas exist across a wide variety of habitat types and altitudinal ranges where sleeping cliffs are available with variable levels of habitat degradations and alterations. They inhabit near human settlement areas where agricultural activities are intense. As the result of habitat losses throughout their geographical ranges geladas currently occupy ~10% of their original habitat (Gippoliti, 2010). Competition from domestic livestock has forced the geladas to remain on the less productive gorge slopes (Abu et al., 2018; Kifle et al., 2013). Since the habitats of geladas are occupied by humans and their livestock, the availability of grazing pastures are decreasing from time to time. In addition, geladas raid cereal crops, resulting in potential conflict with local farmers, and they are continually harassed during crop growing months (Kifle & Bekele, 2020a). Thus, because of such ongoing expansion of subsistence farming, human settlement, competition for grazing pasture, and conflict with local farmers, they are vulnerable to future decline and local extinction (Bergman & Beehner, 2013).

In addition to habitat disturbances and degradations by human agricultural and grazing practices, seasonal variation in food availability also influences the feeding ecology of geladas (Fashing et al., 2014; Jarvey et al., 2018). So far, the feeding ecology of gelada populations in human‐disturbed habitat in unprotected areas has not been intensively investigated. In addition, most of the previous studies of gelada feeding ecology have been carried out in Afroalpine protected ecosystems (Dunbar, 1977; Fashing et al., 2014; Hunter, 2001; Iwamoto, 1979; Jarvey et al., 2018; Woldegeorgis & Bekele, 2015). However, only a few brief (lasting a few weeks to a few months each) studies of gelada feeding ecology have been conducted in human‐modified landscapes of unprotected areas (Abu et al., 2018; Dunbar & Dunbar, 1974; Kifle et al., 2013). Thus, little is known about gelada feeding ecology living in human‐dominated habitats. Therefore, we carried out this long‐term intensive behavioral study on the feeding ecology of southern geladas in a human‐degraded unprotected Afromontane habitat, Wollo, north‐central Ethiopia. Understanding the feeding ecology of primates in human‐modified habitats will contribute for understanding how changing environments shape primate ecology and evolution (Jarvey et al., 2018) and their capacity to coexist in the long term with their human neighbors (Hill, 2017). Thus, by studying the diet of southern geladas in human‐modified habitats, we can understand their ecological and behavioral adaptations in disturbed environment. The objectives of this study were (a) to provide feeding ecology data on a band of southern geladas inhabiting human‐modified habitat; (b) to examine seasonal variation in the diet composition of the specified primate; and (c) to investigate how the level of green grass food availability affect the diet choice of geladas.

## 2 METHODS

### 2.1 Study site and habitat characteristics

**FIGURE 1:**
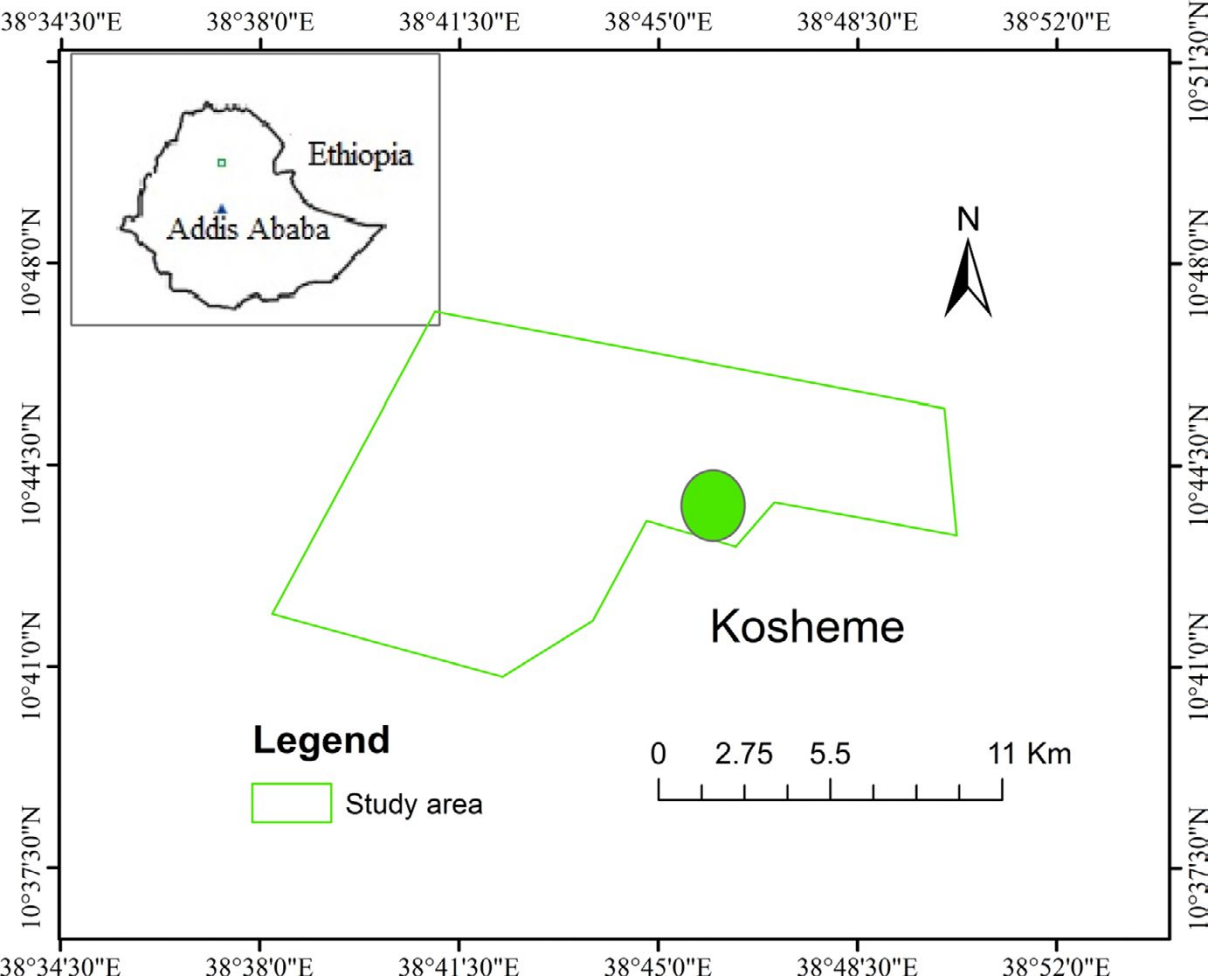
Map showing location of the study area in Ethiopia

The study was carried out at Kosheme near Mekanselam town, the town of Borena *Woreda* (local administrative district), Wollo, Ethiopia. Its geographical location lies at latitude 10°43′51.90″N and longitude 38°47′2.32″E (Figure 1). It is part of the watershed area of Yeshum River, the tributary of Abbay River. The altitudinal range of the area is 1,560–2,500 m a.s.l. This region is an Afromontane grassland ecosystem, and the area is unprotected, where the local people use it for human settlements, farmlands, and livestock pastures. However, in recent years, watershed conservation activities have been initiated by the local communities to protect and rehabilitate the remnant trees, shrubs, and bushes as well as grasslands of the area. Kosheme consists of rocky escarpments, steep cliffs and gorges, valleys with sparse tree cover, shrublands, and strips of grassland plateau. The area possesses Afromontane vegetation like *Acacia* spp. *Ficus* spp, *Rhus* spp, trees, and different shrub, herb, and grass species.

Beside southern geladas, the study area supports several species of mammals including olive baboons (*Papio anubis*), grivet monkeys (*Chlorocebus aethiops*), spotted hyenas (*Crocuta crocuta*), leopards (*Panthera pardus*), African wolves (*Canis aureus*), serval cats (*Felis serval*), Cape hyraxes (*Procavia capensis*), klipspringers (*Oreotragus oreotragus*), crested porcupines (*Hystrix cristata*), mongooses (*Herpestes* spp.), and hares (*Lepus* spp.).

**FIGURE 2:**
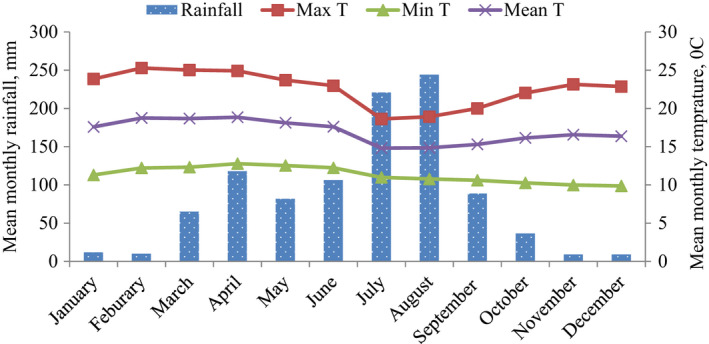
Mean daily maximum, minimum, and average temperatures, and mean cumulative monthly rainfall at Mekaneselam from January 2007 to December 2016 (data source: National Meteorological Agency, Ethiopia, Addis Ababa, 2017)

The area experiences two main seasons: the wet season and the dry season. The wet season typically occurs from June to September, while the dry season occurs from October to May. Occasionally, there is a short rainy period in January, March, and April. This small rain is erratic and highly variable. Temperature and rainfall data for Kosheme were taken from Mekaneselam Meteorological Station (Figure 2), about 2.5 km away from the home range of the study band. The mean annual maximum and minimum temperature was 22.6℃ and 11.3℃, respectively, while mean annual rainfall was 1,004.3 mm over 10‐year period (2007–2016).

### 2.2 Study population

We selected a band of southern gelada populations for detailed behavioral study. Since this study was the first at Kosheme, we habituated the band to human observers for 3 months (from February to April 2015) by following the band from dawn to dusk. We confirmed habituation of the selected band when all fleeing and defensive behaviors disappeared, and travel and feeding activities took place in a relaxed manner as well as when the band tolerated us at a distance of 5–10 m. The group size of the band was 37 individuals on average.

### 2.3 Feeding ecology

During the 18‐month period (May 2015–March 2017), we collected activity budget data from individuals using instantaneous scan sampling (Altmann, 1974). After habituation period, we collected the first 12 months behavioral data every month and the last 6 months data on bimonthly basis. We collected the behavioral data for 5 days in each month at 15‐min intervals for up to 5 min duration by following the band during the daylight hours (Mekonnen et al., 2017). During each scan, we collected activity data from the first 5 random individuals (adults or juveniles but not from infants) in order of occurrence from left to right that avoid possible biases toward eye‐catching activities, recording the first activity they engaged in that lasted ≥3 s (Fashing et al., 2014). We began behavioral data collection, when the band left the sleeping cliff and climbed to its top in the early morning (typically at 07:00 hr). Then, we followed the band throughout the daylight hours until individuals returned back to their sleeping cliff (typically at 18:00 hr). Monthly sampling effort was evenly distributed throughout the study period. At each scan interval, when an individual gelada was observed feeding, we recorded the type of diet item and growth form (Di Fiore, 2004; Fashing et al., 2014; Jarvey et al., 2018; Mekonnen et al., 2018). We defined feeding behavior as picking, grazing, handling, foraging, chewing, excavating, or manipulating any potential food items. If an individual gelada was handling multiple diet items during a particular scan, we considered the most abundant food item in the hand to be the diet item. We categorized the food items as grasses (blades, rhizomes, seeds, corms, or bulbs), herbs (leaves, flowers, roots), shrubs (flowers, fruits, or buds), trees (fruits, piths, seeds, or gums), invertebrates (ants, termites, or alates), or others (Dunbar, 1977; Fashing et al., 2014; Iwamoto, 1979) as well as crops (grains, seedlings, or vegetative).

### 2.4 Monitoring temporal patterns of food availability

We monitored phenological data on the grass and herb greenness levels to evaluate patterns of temporal change in food availability at monthly intervals over the study period (Fashing et al., 2014; Hunter, 2001; Jarvey et al., 2018). We collected the phenological data from the randomly selected permanent plots (each 50 × 50 cm). We constructed 22 plots within the home range of the study band to follow up the levels of greenness and desiccation of herbs and grasses using visual inspections. Depending upon their patterns of temporal greenness changes, we assigned a score for each plot from 0–3, where 0 = 0% (absence of green grass or/and herb within a particular plot), 1 = <33% (brown grass or/and herb with slight green of a particular plot), 2 = 31%–66% (light green grass or/and herb), and 3 = ≥67% (strictly green grass or/and herb) after detailed inspections. Monitoring of each plot was tightly correlated with behavioral observations of the study periods.

### 2.5 Data analysis

We calculated the contribution of each food item to the total diet consumption using the proportion of the total number of feeding records spent on each diet type. We summed the daily food item consumptions within each month to construct monthly proportion of diet item composition. We then calculated mean seasonal and overall dietary composition by averaging the monthly percentage proportions. We used the following formula to calculate the availability of green grass and/or herb: monthly greenness level = (greenness score of a plot × n + …)/N, where *n* = number of plots that scored a particular greenness level; *N* = total number of plots examined per a month. For seasonal analysis, we included data from October to April as dry season and from May to September as wet season. Although May typically is known as a dry season in the area, we included both May 2015 and 2016 into the wet season as the result of unusually heavy rainfall during the study period.

### 2.6 Statistical analysis

We conducted all statistical tests using SPSS version 20 software (IBM SPSS Inc., Chicago, USA). We used Shapiro–Wilk tests to test for normality and Levene tests to check homogeneity of variances (*p* > .05). We used a one‐way ANOVA model to test for differences in the percentage consumption of each growth form and diet item between seasons. When the assumptions of parametric tests on the proportional food item did not meet, we performed arcsine square root transformations prior to statistical analysis to fulfill ANOVA model assumptions (Sokal & Rohlf, 1981). We tested the correlation between mean monthly green grass availability and the monthly percent feeding time of each diet category using a Pearson's rank correlation test. We set the level of statistical significance at *p* ≤ .05 for all analyses.

## 3 RESULTS

### 3.1 Monthly diet

We recorded a total of 21,135 activity scans (10,471 recorded as “feeding” over 1,057 hr across 90 days. Feeding accounted for 49.5% of the band's overall activity time budget (Kifle & Afework in prep). Southern geladas exhibited wide variability in their diet item consumptions across months (Table 1). Grass blades constituted the bulk for southern gelada's diet throughout the study months. The monthly proportion of consumption of grass blades ranged from 21.4% to 97.5%. The band also devoted its feeding time for grass rhizomes 0.0%–36.3%, grass seeds 0.0%–47.3%, and grass bulbs 0.0%–28.7% in monthly proportion of diet consumption. The band consumed considerable amount of food items from tree and bush parts, fruits ranging from 0.0% to 29.6% and flowers 0.0%–2.8% in the monthly proportion of consumption. In addition, southern geladas at Kosheme added a considerable amount of crop grains, seedlings, and its vegetative parts (0.0%–41.5%). Similarly, geladas ate invertebrates especially termite alates, each June when they appeared in mass shortly just on the onset of the rainy season. Occasionally, the band also fed on other (limestone), possibly for mineral intake.

**TABLE 1:** Percent monthly diet item contribution for southern geladas at Kosheme, Wollo, Ethiopia, from May 2015 to March 2017

Month	*N*	Monthly contribution, %												
		Grass					Herb			Tree/shrub				
		BL	RH	CO	SE	BU	HL	HF	HR	FR	TSA	CR	IN	OA
May 2015	512	97.5	0.0	0.0	0.0	0.0	2.2	0.0	0.0	0.0	0.4	0.0	0.0	0.0
Jun 2015	457	92.8	0.0	0.0	0.0	0.0	6.1	0.0	0.0	0.0	0.7	0.0	0.4	0.0
Jul 2015	420	63.8	0.0	0.0	0.0	0.0	1.4	0.0	0.0	0.0	0.0	33.8	1.0	0.0
Aug 2015	528	72.5	0.0	0.0	0.0	7.2	11.6	0.0	0.0	0.0	1.9	5.9	1.0	0.0
Sep 2015	497	33.8	0.0	0.0	47.3	4.8	10.3	2.8	0.0	0.0	0.8	0.0	0.2	0.0
Oct 2015	663	42.4	7.1	0.3	10.7	28.7	7.1	0.2	0.0	0.0	1.8	1.4	0.5	0.0
Nov 2015	496	44.0	3.2	0.2	3.6	0.0	4.4	1.2	0.0	0.6	1.0	41.5	0.2	0.0
Dec 2015	639	57.0	3.0	0.8	0.0	3.9	0.5	0.0	0.0	29.1	0.3	5.2	0.3	0.0
Jan 2016	544	49.3	19.1	0.4	0.7	5.7	3.7	0.0	0.0	17.5	3.7	0.0	0.0	0.0
Feb 2016	665	39.6	35.3	8.7	0.0	13.2	2.0	0.0	0.5	0.2	0.5	0.0	0.2	0.0
Mar 2016	671	76.2	15.7	1.9	0.0	3.0	0.8	0.0	0.3	0.6	1.3	0.0	0.3	0.0
Apr 2016	709	30.2	36.3	6.4	0.0	11.3	1.8	0.0	1.6	7.3	4.7	0.0	0.6	0.0
May 2016	691	86.3	0.0	0.0	0.0	0.7	3.6	0.0	0.1	5.1	3.6	0.0	0.3	0.3
Jul 2016	627	86.8	0.0	0.0	0.5	0.0	5.4	0.0	0.0	0.0	2.4	1.1	3.4	0.5
Sep 2016	564	39.9	0.0	0.0	38.3	0.9	6.0	11.4	0.0	0.0	2.7	0.7	0.2	0.0
Nov 2016	617	42.5	12.0	1.0	2.6	4.2	2.4	1.5	0.8	19.3	1.0	12.5	0.0	0.3
Jan 2017	533	21.4	25.5	3.8	0.0	1.9	2.4	0.0	0.4	29.6	0.8	14.3	0.0	0.0
Mar 2017	638	31.2	28.4	8.0	0.0	7.7	2.4	0.2	1.1	15.5	4.9	0.0	0.5	0.3

Abbreviations

Key: BL=blade; RH=rhizome, SE=seed, CO=corm, BU=bulb, HR=herb root, HF=herb flower, HL=herb leave, FR=fruit, TSA=other part of tree and shrub includes piths, buds, seeds, flowers, and gums, CR: crop (grain, seedling, and vegetative), IN: invertebrate, OA: other. *N* = number of records

### 3.2 Relationship between green grass availability level and diet item consumption

Monthly green grass/herb availability level was significantly positively correlated with monthly grass blade (*r* = 0.647, *p* = .004) and herb aboveground (*r* = 0.526, *p* = .025) consumption. However, monthly green grass/herb availability (*N* = 18) was significantly negatively correlated with monthly underground grass (*r* = −0.825, *p* < .001), herb underground (*r* = −0.825, *p* = .003), and tree/shrub part (*r* = −0.825, *p* =.003) consumption. The consumption of grass seed, crop and invertebrate had no significant relationship with green grass/herb availability (grass seeds: *r* = 0.349, *p* = .155; crop: *r* = 0.006, *p* = .081; invertebrate: *r* = 0.342, *p* = .165).

During the 18 months, when southern geladas consumed heavily on grass blades, there was reduction in the consumption of underground grasses and herbs. Grass blade consumption was significantly negatively correlated with monthly underground grass item (*r* = ˗0.623, *p* = .006) and herb underground (*r* = ˗0.479, *p* = .004) consumption. However, monthly (*N* = 18) grass seed consumption was significantly positively correlated with monthly herb aboveground (*r* = 0.802, *p* < .001) consumption. In addition, underground grass item consumption was significantly positively correlated with monthly herb underground (*r* = 0.717, *p* = .001).

### 3.3 Overall diet

Grasses accounted for the largest part of the overall diet for southern geladas. Out of the total plant growth forms, grass parts cumulatively comprised 79.6% of the overall monthly average diet of southern geladas at Kosheme. From the grass parts, blades contributed 55.4%, rhizomes 11.2%, seeds 5.4%, corms 2.0%, and bulbs 5.6% of the overall diet. Herb parts cumulatively accounted for 5.2% of the overall diet (leaves: 4.0%, flowers: 0.9%, roots: 0.3%). Tree parts comprised 8.0% of the overall diet (fruits: 7.1%, piths: 0.3%, gums: 0.3%, seeds: 0.3%), and shrub parts comprised 1.1% (flower: 0.5%, fruits: 0.2% and buds: 0.4%), and cultivated cereal crops accounted for 5.6% of the overall diet. Additionally, invertebrates comprised 0.5% and other items 0.1% of the overall diet.

From the overall total 8,332 grass diet records, blades comprised 69.6%, rhizomes 14.1%, seeds 6.8%, corms 2.4%, and bulbs 7.1% of the overall diet. Similarly, from the overall total of 542 herb diet records, leaves comprised 76.8%, flowers 17.5%, and roots 5.7% of the overall diet. From the total of 839 tree feeding records, fruits accounted for 88.0%, seeds 4.3%, piths 3.9%, and gums 3.8%. At Kosheme, fruits from trees contributed 98.1%, and 1.9% from shrubs for southern gelada diet. The fruit of *Ficus sycomrous* accounted for 84.8% from a total 738 tree fruit records and 83.2% from overall 752 fruit records. In addition, from the total of 112 feeding records of shrubs, flowers accounted for 47.3%, buds 40.2%, and fruits 12.5%. Southern geladas at Kosheme also raided cereal crops. From the total of 585 cereal crops (bean: *Vicia faba*, wheat: *Triticum* spp, teff: *Eragrostis tef*, chickpea: *Cicer arietinum,* chickling vetch: *Lathyrus sativus*) consumption records, grains at the time of sowing comprised 23.9%, seedling 7.5%, crop heads at the time of vegetative/fruiting stages 4.9%, and leftover grains at the time of sawing/harvesting 63.6% of the overall diet.

### 3.4 Seasonal variation in the feeding ecology of southern geladas

Considering the overall food source/growth form categories, geladas consumed more grass parts during the wet season than the dry season; however, the difference was not significant (84.1 vs. 74.8%, *F*_1_,_16_ = 1.87, *p* = .191). The band also spent more time to consume herb parts during the wet season than the dry season, and the difference was significant (7.6 vs. 3.5%, *F*_1_,_16_ = 4.41, *p* = .052). Similarly, there was no significant difference in food sources from shrub parts between the wet (1.4%) and dry (0.7%) seasons (*F*_1,16_ = 2.25, *p* = .153). Food sources from tree parts accounted for 13.3% and 0.8% of the wet and dry seasons diet, respectively, and the difference was significant (*F*_1,16_ = 8.38, *p* = .011). Cereal crop accounted for 5.2% of the wet season diet and 7.5% of the dry season; however, the difference was not significant (*F*_1,16_ = 0.15, *p* = .704).

**FIGURE 3:**
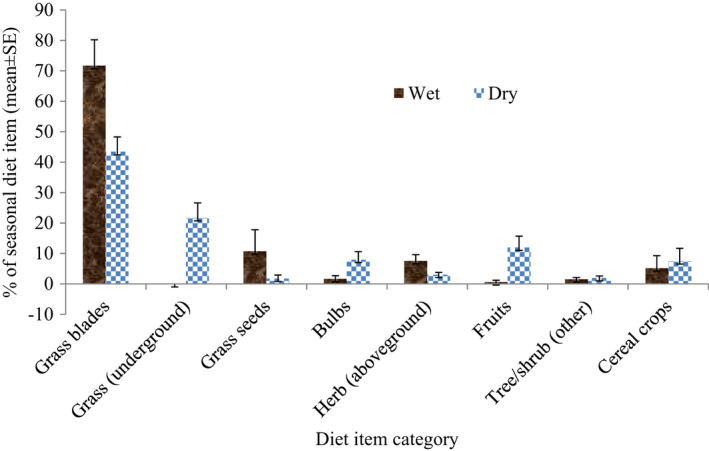
Mean seasonal percent of time spent feeding on different food items by southern geladas at Kosheme, Wollo, Ethiopia. Values represent the mean ± SE; grass underground includes grass corms and rhizomes; herb aboveground represents leaves and flowers of herbs; tree/shrub (other) represent piths, leaves, buds, seeds, flowers, and gums of trees and/or shrubs

Considering the food item consumption categories, the diet of southern geladas varied markedly across the two seasons (Figure 3). The band shifted its diet item consumptions from more grass blades during the wet season to a much greater dependence on the underground diet item during the dry season. Grass blades accounted for a significantly greater proportion of the diet during the wet season than the dry season (*F*_1,16_ = 9.21, *p* = .008). Grass seeds contributed 10.8% of the wet season and 1.8% of the dry season, but the difference was not significant (*F*_1,16_ = 2.00, *p* = .176). There was no contribution of rhizome and corm diet items during the wet season. However, geladas at Kosheme consumed 18.6% rhizomes and 3.2% corms during the wet season (rhizomes: *F*_1,16_ = 17.49, *p* = .001; corms: *F*_1,16_ = 6.96, *p* = .018). Thus, the contribution of underground grass parts was nil during the wet season but 21.7% during the dry season, and the difference was significant (*F*_1,16_ = 15.45, *p* = .001). Similarly, bulbs comprised 1.7% of the wet season and 8.0% of the dry season, but the difference was not significant (*F*_1,16_ = 4.08, *p* = .060). Aboveground herb parts (leaves and flowers) contributed 7.6% of the wet season and 3.1% of the dry season for the band, and the difference was significant (*F*_1_,_16_ = 5.36, *p* = .034). On the other hand, southern geladas at Kosheme ate herb roots 0.01% during the wet season and 0.5% during the dry season, and the difference was significant (*F*_1_,_16_ = 5.49, *p* = .032). Similarly, fruits accounted for 0.6% of the wet season and 12.0% of the dry season diet, and difference was significant (*F*_1_,_16_ = 7.10, *p* = .017). The band spent more time feeding on invertebrates during the wet season (0.8%) than the dry season (0.3%); however, the difference was insignificant (*F*_1_,_16_ = 2.40, *p* = .141). Finally, the contribution of tree/shrub part (other) was 1.6% during the wet season and 2.0% during the dry season (*F*_1_,_16_ = 0.35, *p* = .563).

## 4 DISCUSSION

Only a few studies have been carried out on the feeding ecology of geladas in unprotected areas of human‐disturbed habitats, and these studies have been conducted for short duration that lasted a few weeks or few months (Abu et al., 2018; Dunbar & Dunbar, 1974; Iwamoto & Dunbar, 1983; Kifle et al., 2013). Thus, the feeding ecology and diet composition of geladas inhabiting unprotected landscapes remains largely unstudied. Therefore, this long‐term intensive study was conducted to describe the feeding ecology and diets of southern geladas inhabiting a disturbed environment and to quantify seasonal differences in the diet along north‐central highlands of Ethiopia.

At Kosheme, a human‐disturbed unprotected afromontane habitat in north‐central Ethiopia, we found that the overall diet of southern geladas consisted of 79.6% grass parts, 5.2% herb parts, 8.0% tree parts, and 1.1% shrub parts. In addition, the diet of southern geladas comprised 5.6% cereal crops and 0.5% invertebrates. We also found that the diet of southern geladas at Kosheme was diverse and showed wide variability across months and seasons. Grass blades and herb leave consumption correlated positively, and underground food items consumption correlated negatively, with monthly level of green grass/herb availability. Our result offers insights into the dietary strategies southern geladas adopt to cope with human‐modified landscapes of the north‐central Ethiopian Highlands.

In general, previous studies showed that geladas primarily consume greater proportions of grass blades (Dunbar, 1977; Fashing et al., 2014; Hunter, 2001; Iwamoto, 1993; Jarvey et al., 2018; Woldegeorgis & Bekele, 2015). In line with this, the overall diet for geladas at Kosheme consisted of 55.4% grass blades followed by underground grass items (rhizomes, corms, and bulbs: 18.8%) and fruits (7.3%). However, the proportions of gelada diets are highly variable across study sites (Table 2). For example, Fashing et al. (2014) found that gelada diet comprised 50.6% grass blades and 28.6% herb leaves at Guassa. Similarly, Iwamoto (1979) found that geladas consumed 68.8% grass blades and 15.7% herb leaves at Gich, Simien Mountains National Park. In contrast, herb leaves made up 4.0% of the overall diet of geladas at Kosheme, and 7.3% at Sankaber, Simien Mountains National Park (Jarvey et al., 2018). Similarly, geladas at Kosheme ate 7.3% fruits, and 3.6% at Indetu, Arsi. However, geladas at Guassa (Fashing et al., 2014) and at Gich (Iwamoto, 1979; Woldegeorgis & Bekele, 2015) did not include any fruit in their menu. In a similar finding, geladas at Kosheme also ranged through crop fields and fed on considerable amounts of grain (5.6%) at the sowing time, and fallen grain from the previous harvest. However, Fashing et al. (2014) and other studies in different sites showed that geladas did not include any grain from cereal crops.

**TABLE 2:** Overall mean gelada diets across the study sites

Study site	Elevation (m)	No. of study months	Percent of overall diet									
			Grass blades	Grass seeds	Grass underground	Herb aboveground	Herb roots	Fruits	Tree/shrub others	Invertebrates	Crops	Others
Kosheme, Wollo^1^	2,400	18	55.4	5.4	18.8	4.9	0.3	7.3	1.9	0.5	5.6	0.1
Guassa, Menz^2^	3,450	15	50.6	2.2	4.0	29.1	7.5	0.0	0.0	2.8	0.0	3.8
Sankaber, Simien mts^3^	3,300	6	55.2	1.8	7.6	6.2	20.5	3.3	0.0	0.1	0.0	1.1
Sankaber, Simien mts^4^	3,300	5	45.0	23.2	24.5	2.5	1.4	1.0	0.0	0.1	0.0	2.3
Sankaber, Simien mts^5^	3,300	12	76.3	1.3	14.0*	7.3	‐	0.5	0.0	0.1	0.3	0.2
Gich, Simien mts^6^	3,900	3	68.8	5.1	8.0	15.7	2.5	0.0	0.0	0.0	0.0	0.0
Gich, Simien mts^7^	3,900	12	74.3	0.6	7.6	5.2	10.6	0.0	0.0	0.0	0.0	1.4
Bole, Debre Libanos^8^	2,300	6	91.4	5.0	0.5	0.6	0.0	2.0	0.0	0.0	0.0	0.5
Indetu, Arsi^9^	2060	9	51.7	0.0	26.1	7.1	8.7	3.6	0.0	0.0	0.0	3.0

^1^ This study.

^2^ Fashing et al., 2014.

^3^ Hunter, 2001.

^4^ Dunbar, 1977.

^5^ Jarvey et al.,2018 where * includes both underground grass and herb diet items.

^6^ Iwamoto, 1979.

^7^ Woldegeorgis & Bekele, 2015.

^8^ Dunbar & Dunbar, 1974.

These differences in gelada diet across study sites may be attributed to several factors. The length of studies (most previous studies were shorter in duration, ranging from 3 to 9 months, missing the full annual variation of ecological conditions of the year) and variations in the rainfall pattern, elevation, and human disturbance may influence dietary differences across studies. Study duration or seasonally biased sampling may account for differences on food item consumptions and compositions among monkey populations (Xiang et al., 2012). Studies also showed that human disturbance and land use severely affect primate diets (Fashing et al., 2014; Hunter, 2001; Irwin, 2008; Riley, 2007; Singh et al., 2001). Tesfaye et al. (2021) also found that Omo River guereza (*Colobus guereza guereza*) ate considerable dietary variability among populations experiencing different degrees of habitat fragmentation and degradation. Similarly, Bale monkeys that inhabited fragment habitats modified their diet by increasing consumption of fruits, stems, petioles, and insects other than bamboo leaves (Mekonnen et al., 2018). In addition, the availability of a particular diet item at a particular site is likely to be a primary contributor to the variability in the diets of geladas across study sites. For example, elevation and habitat composition in terms of vegetation types influence the diet of colobine monkeys (Bennett & Davies, 1994) and gray snub‐nosed monkeys (Xiang et al., 2012). In Ethiopia, habitats at relatively lower elevations (Afromontane ecosystem e.g., Kosheme) tend to be more floristically diverse and typically contain a wider range of food types available to geladas when compared to habitats at higher elevations (Afroalpine ecosystem e.g., Gich, Sankaber and Guassa).

Studies revealed that primates in disturbed forest habitats consumed less diverse diets than intact habitats (Riley, 2007; Tesfaye et al., 2013). However, geladas are unique among primates in that they permanently inhabit the grassland habitats of the Ethiopian Highlands, and the impact of human disturbances on the diets of this primate species inhabiting such open grassland habitats remains unstudied. Our current study revealed that southern gelada populations at Kosheme consumed large varieties of diet items in different proportions. Such consumption of diverse food items is one of the dietary strategies of southern geladas to persist in human‐modified landscapes across the Ethiopian Highlands. Fashing et al. (2014) pointed out that geladas at Guassa, an intact Afroalpine ecosystem, consumed a much more varied diet than geladas at more human‐dominated sites. However, contrary to the study of Fashing et al. (2014), the present work revealed that geladas that inhabit in human‐modified landscape habitats consumed more varieties of diet items than geladas at other study sites (Table 2), likely reflecting more diverse food availability due to favorable climate condition of the Afromontane ecosystem at Kosheme. Thus, the current study pointed out that Afromontane habitats likely to promote geladas to consume more diversified diet items upon their diverse availability than geladas living in Afroalpine ecosystems like Guassa (Fashing et al., 2014), Gich (Woldegeorgis & Bekele, 2015), and Sankaber (Hunter, 2001; Jarvey et al., 2018). On the other hand, as the preferred food items decline in human disturbance habitats, primates feed more widely on less preferred foods species, which are easier to obtain in the area (Chaves et al., 2012; Dela, 2007; Dunn et al., 2012; Grassi, 2006).

Geladas, like other primates, are characterized by seasonal changes in diet throughout the year (Fashing et al., 2014; Jarvey et al., 2018). Our study also showed that southern geladas at Kosheme exhibited wide variability in diet item consumptions across months and seasons likely to reflecting temporal variations in particular food availability during those periods. Although geladas are predominant graminivore, they show dietary variability associated with seasonal declines in grass leaf availability (Dunbar, 1977; Hunter, 2001; Iwamoto, 1979; Jarvey et al., 2018). For example, studies on gelada populations at Guassa (Fashing et al., 2014) and in the Sankaber region of the Simien Mountains National Park (Jarvey et al., 2018), the time geladas spent consuming grass blades decreased when green grass blade availability was low and the time spent consuming underground foods increased. In line with this, we found that the diet of southern geladas at Kosheme was highly inclined on grass blades consumptions, which make up 71.7% of the consumed diet items during the wet season compared to 43.4% during the dry season when green grass blade availability became low.

At the beginning of the wet season, freshly growing grass blade consumptions by southern geladas increased sharply when grasses recovered rapidly from dryness due to the presence of rainfall. Other studies also showed that geladas appear to prefer green grass blades during the wet season (Dunbar, 1977; Fashing et al., 2014; Hunter, 2001; Iwamoto, 1979, 1993; Jarvey et al., 2018). For example, during the wet season, grass blades accounted for >90% of the diet at different gelada study sites (Bole: Dunbar & Dunbar, 1974; Sankaber: Dunbar, 1977 and Hunter, 2001; Gich: Iwamoto, 1993). Such higher consumption of grass blades might be due to their easy availabilities and higher nutritional qualities during the wet season compared to the dry season. Temporal variation in food availability is one of main factors determining seasonal variation in the diet of primates (Hanya, 2004; McConkey et al., 2003; Poulsen et al., 2001). Grass blades consumption also varied among wet season months (maximum 97.5% and minimum 33.8%) at Kosheme and maximum 93.2% and minimum 80.2% at Sankaber (Jarvey et al., 2018). Such monthly variation in grass blade consumptions might be due to young grass blade availability at the beginning of wet season months, and maturity of grass blades and switch on grass seed consumption at the end of the wet season months.

Seasonal dietary shifts are a common feature of primates (Guo et al., 2007; Li, 2006; Xiang et al., 2007). Southern geladas at Kosheme did not include underground grass items (rhizomes and corms) in their wet season diet. However, they shifted to consume these diet items during the dry season, when the green grass blade scarcity reached at maximum level. Such underground diet items accounted for 21.7% on average during the dry season, and up to 57.7% of the dry season months for southern geladas at Kosheme, Wollo, Ethiopia. Similar studies also showed that underground diet items comprised a larger part of the dry season diet than the wet season for geladas (e.g., Jarvey et al., 2018). Primate diets are severely affected by seasonal variation in preferred food availabilities (Irwin, 2008; Jarvey et al., 2018; Riley, 2007). Thus, when the availability of the preferred green grass blades decreased during the dry season, southern geladas at Kosheme increased underground food item consumption. Study suggested that underground foods are fallback foods for geladas (Fashing et al., 2014; Jarvey et al., 2018), and the consumption of underground food does not appear to be influenced by availability, but rather by the lack of preferred diet items (Altmann, 2009; Marshall & Wrangham, 2007). When green grass availability remains higher during the dry season, underground food items constitute less of the dry season diet (Jarvey et al., 2018).

Iwamoto and Dunbar (1983) and Jarvey et al. (2018) at Sankaber, Zewdu et al. (2013) at Wonchit Valley, and Fashing et al. (2014) at Guassa noted that the consumption of underground food items increased during the dry season and the consumption of such underground items exhibited significant negative correlations with rainfall (Fashing et al., 2014; Jarvey et al., 2018). The present study also suggests that the scarcity of green grass blades might be the reason for shifting the consumption of underground food items during the dry season when the rainfall reached minimum level. During the dry season, when the green grasses dry out, geladas can shift their foraging profile to digging for more subterranean food items (Hunter, 2001). Such subterranean plant parts like tubers provide an alternative source of energy in the form of carbohydrates (Byrne et al., 1993). Likewise, addition of underground diet items like bulbs and rhizomes by geladas for their diet menu may be good sources of carbohydrates, proteins, and other nutrients during the dry season.

Exploration of underground diet items represents an adaptation that allowed all the *Theropithecus* to tap subterranean storage food sources that are unavailable to the gelada's main competitors, namely wild ungulates (Dunbar & Bose, 1991). Similarly, in human‐dominated landscapes, livestock and pack animals are the main competitors of geladas on the aboveground grass items at Kosheme (Kifle, personal observation). Geladas rely more heavily on underground foods in habitats more heavily influenced by humans (Fashing et al., 2014; Jarvey et al., 2018).

Higher consumption of fruits from trees and shrubs is another interesting finding of this study to understanding the dietary flexibility of southern geladas in human‐disturbed habitats. During the dry season, the contribution of fruits for geladas diet at Kosheme was high. For example, they consumed fruits up to 29% in some months. This finding is incongruent with studies that argued geladas to be obligate graminivores (Dunbar, 1977; Dunbar & Dunbar, 1974; Iwamoto, 1993; Iwamoto & Dunbar, 1983; Jarvey et al., 2018). The main sources of fruit for geladas diet at Kosheme were *Ficus sycomorus* trees. Fruits from *Rosa abyssinica* are also good sources of diet item at Sankaber (Hunter, 2001). The fruit of *Ficus* species contains excess amount of sugar (Byrne et al., 1993) and are known to be keystone species that help sustain frugivores (Byrne et al., 1993). Thus, fruits from *F*. *sycomorus* might be an important diet item that provides relief for the nutritional bottleneck during the dry season for southern geladas at Kosheme. In turn, southern geladas may contribute to disperse the seeds of *Ficus* spp. over long distances, away from the mother tree as these seeds are not easily digested in the alimentary canal of geladas (Kifle, personal observation). *Ficus* spp. also occurred in scattering patterns at Kosheme, nonetheless, the band repeatedly visited these trees every day in the early morning during the fruiting months (Kifle, personal observation). Our study band traveled long distance from their sleeping site to consume the fruits.

Many primate species exhibit a tendency of consuming crops from the surrounding farmlands (Hill, 2017; Kifle & Bekele, 2020a, 2020b; Seiler & Robbins, 2016; Tweheyo et al., 2005). For example, Bale monkeys that inhabit fragmented habitats are engaged in crop raiding (Mekonnen et al., 2018). Likewise, southern geladas at Kosheme are engaged in crop raiding and consumed cereal crops during the time of sowing, vegetative, fruiting, and harvesting stages as well as on fallen grain from the previous harvested farmlands. Most of feeding on cereal crops occurred just immediately harvesting on those fallen grains and at the time of sowing when the local farmers are reluctant at this time as they assumed that geladas do not get grains from the soil. At Kosheme, geladas consumed cereal crops opportunistically, because they are typically more nutritious, since agricultural activities occur during both wet and dry seasons in the region. Farmer responses to crop raiding by southern geladas included shouting, stoning, bouldering, sticking, slinging, hunting with spears, chasing them with or without dogs, or positioning scarecrows (Kifle & Bekele, 2020a).

The overall consumption of cereal crops by geladas at Kosheme accounted for 5.6%, which is one particular intriguing finding in human‐disturbed habitats of this study. Jarvey et al. (2018) pointed out that geladas at Sankaber in the Simien Mountains National Park added only 0.3% of cereal crops in their annual diet. Fashing et al. (2014) did not record any crop item consumed by geladas during their 15‐month study period at Guassa (Table 2). Extreme constriction and degraded environment of unprotected landscape reinforce gelada populations range to include more areas of human use that may promote them to consume more cereal crops from the nearby farm fields. Cereal crops are more nutritious and help geladas to survive in the severely modified environment even if crop raiding has a greater risk up to death by local farmers. Many primates that lost their preferred habitats feed on cereal crops to increase their foraging efficiency and nutrient intake (Naughton‐Treves et al., 1998). Likewise, many primates in agricultural mosaic landscapes often supplement their diet by consuming crops (Anderson et al., 2007; Chaves & Bicca‐Marques, 2016; Maibeche et al., 2015), leading to potentially negative encounters with farmers (Bryson‐Morrison et al., 2017).

## 5 CONCLUSION

Anthropogenic habitat disturbances can dramatically affect the quality, availability, and distribution of food resources and the addition of anthropogenic food sources into the diets with both positive and negative effects on the survival of primates (Higham et al., 2009; Hoffman & O' Riain, 2010; Mekonnen et al., 2018; Tesfaye et al., 2021). This study contributes to our understanding of the ecological and dietary flexibility of primates in human‐modified environments, as well as conservation implication of such flexibility. In addition, determining how primate diets, such as that of geladas, shift in response to human disturbances is important to understanding how changing environments shape primate ecology and evolution (Hill, 2017; Jarvey et al., 2018). The results of this study shows that geladas inhabit human‐disturbed environment consumed more diverse diet items. The consumptions of such diversified diet items are the major dietary strategies of geladas to survive in human‐modified landscapes. Similar study on the Bale monkeys (*Chlorocebus djamdjamensis*) in southern Ethiopia showed that those groups that inhabit fragmented landscapes exploited far more plant species to broadening their diet than conspecific in continuous forest (Mekonnen et al., 2018). Southern geladas can respond to anthropogenic habitat disturbances by incorporating more alternative food items such as cereal crops, fruits, bulbs, roots, rhizomes, corms, piths, buds, and seeds. Such dietary flexibility in the consumptions of these food items is key factors for the survival strategies of geladas in human‐modified environments.

Our results also indicate that southern geladas at Kosheme have relied on underground diet items during periods of food scarcity. During the lengthy dry season when green grass changed to brown form, geladas significantly increased their consumption of underground food items at Kosheme. Most of these underground diet items which were consumed during dry seasons consisted of grass rhizomes and bulbs. Similarly, Fashing et al. (2014) pointed out that most of the underground food items which were eaten by geladas at Guassa during the dry season consisted of herb roots through tubers and grass corms and rhizomes. Thus, southern geladas appear to adjust their diets in response to seasonal shortage in their preferred foods (i.e., grass blade). In addition, underground food item and fruit consumptions are the reliable food source for southern geladas during elongated dry season and general food scarcity in human‐disturbed habitats.

Based on the current findings, southern geladas that live in open grassland ecosystem in highly human‐modified landscapes show greater ecological and behavioral flexibility than any other primates. They develop a certain degree of dietary plasticity for living in the devastated environment in Ethiopian Highlands where other primate species cannot cope up to survival by sharing the available food resources (e.g., grasses) with livestock. This dietary flexibility may allow them to persist in areas subject to human influences. In addition, the study concludes that geladas are opportunistic in the diet choices rather than obligate graminivores.

Despite southern geladas show dietary flexibility in human‐modified landscapes, conservation actions in the unprotected areas requires more attention. The need of large foraging areas for geladas should be considered in conservation and management strategies. Thus, leaving mosaic grassland habitats through proper land‐use planning in the human‐dominated landscapes for geladas may alleviate some of the pressures like diminished suitable habitat and food resources. Therefore, effort should be made to the conservation value of landscapes outside the protected areas of the human‐modified habitats for the long‐term survival of geladas and other primates in north‐central Ethiopian Highlands.

## CONFLICT OF INTEREST

None declared.

## AUTHOR CONTRIBUTIONS

**Zewdu Kifle:** Data curation (equal); formal analysis (lead); funding acquisition (lead); methodology (equal); project administration (equal); software (lead); writing–original draft (lead); writing–review and editing (equal). **Afework Bekele:** Data curation (equal); formal analysis (supporting); funding acquisition (supporting); investigation (supporting); methodology (equal); project administration (equal); writing–review and editing (equal).

## Data Availability

Data of this study are available from the corresponding author upon reasonable request.
